# Association between dietary protein intake and frailty index among home-dwelling older adults; an 8-year follow-up study

**DOI:** 10.1016/j.jnha.2026.100850

**Published:** 2026-04-17

**Authors:** Pia Bålsrud, Stine M. Ulven, Anette Hjartåker, Inger Ottestad, Kjetil Retterstøl, Ursula Schwab, Jacob J. Christensen, Magne Thoresen, Kirsten B Holven

**Affiliations:** a Department of Nutrition, Institute of Basic Medical Sciences, University of Oslo, Oslo, Norway; b Clinical Nutrition, Department of Clinical Service, Division of Cancer Medicine, Oslo University Hospital, Oslo, Norway; c The Lipid Clinic, Oslo University Hospital, Oslo, Norway; d Institute of Public Health and Clinical Nutrition, University of Eastern Finland, Kuopio, Finland; e Department of Medicine, Endocrinology and Clinical Nutrition, Kuopio University Hospital, Kuopio, Finland; f Department of Biostatistics, Institute of Basic Medical Sciences, University of Oslo, Oslo, Norway; g Norwegian National Network on Familial Hypercholesterolemia, Oslo University Hospital, Oslo, Norway

**Keywords:** Older adults, Frailty index, Nutrition, Protein, Longevity

## Abstract

**Background:**

The World’s population is getting older, and healthy behavior such as a healthy diet may prevent the development of frailty and promote healthy ageing. In this study, we aimed to investigate the association between dietary protein intake and longitudinal changes in frailty among older, home-dwelling subjects.

**Method:**

Data were obtained from 130 home-dwelling, Norwegian older adults (70+) at baseline and after 8-year follow-up. Frailty was measured by a frailty index (FI) based on an accumulation of 38 deficits, scored 0–1. Dietary protein intake was collected by 2 × 24-h recall per visit. The association between dietary intake at baseline and change in FI was analysed by linear regression modelling. Changes in diet and changes in FI between the two visits were analyzed by Spearman’s rank correlation coefficient.

**Results:**

The FI score significantly increased from baseline to follow-up (0.12 vs 0.2, p < 0.001), and more subjects were categorised as frail at the follow-up visit (3% vs 33%, p < 0.001). Overall, there was no significant association between dietary protein intake at baseline or change in dietary intake and change in FI score. We did, however, observe a decreased intake of protein (E %) and meat (g) after 8-year follow-up.

**Conclusions:**

Our findings suggest that frailty accelerates with age, independent of dietary protein intake. A healthy diet may delay the rate of frailty, however, even if the dietary intake of protein remains stable after 8-year follow-up, it could not prevent the frailty process in our study population. Thus, more studies are needed to describe what factors that might accelerate or prevent the accumulation of frailty deficits with advancing years.

## 1 Introduction

The World’s population is getting older, which emphasizes the importance of healthy ageing [1]. Frailty is strongly associated with ageing which increases vulnerability to stressors and risk of adverse outcomes [2]. Frailty is commonly characterised by Fried et al.’s [3] physical phenotype definition, or by Rockwood et al.’s [4] frailty index (FI). Fried’s physical frailty definition includes five criteria to define frailty: weakness (low grip strength), unintentional weight loss, self-reported exhaustion, slow walking speed, and low physical activity level [3]. Rockwood’s definition is more comprehensive; it measures the accumulation of deficits from different health systems (for example, poor physical function, presence of disease, and psychological parameters such as negative emotions and attitude to own health, and impaired cognitive function), resulting in a continuous FI score [4,5]. The two instruments are very different and should be considered complementary rather than mutually exclusive [6]. In this paper, we used Rockwood’s definition, as this definition is a continuous scale and includes more than only physical parameters.

Adequate nutrition and avoidance of malnutrition improve the quality of life and promote healthy ageing [7]. The prevalence of malnutrition is high in the older Norwegian population [8]. A sufficient intake of energy and protein is necessary to maintain a healthy body weight and body composition, as well as to maintain normal muscle function. Older adults are at higher risk of inadequate protein intake compared to the general population [9]. However, there are few studies on protein intake and frailty using the FI definition. To the best of our knowledge, a very limited number of studies have investigated the association between the intake of protein-rich food groups such as fish, meat, and dairy and the longitudinal changes in FI score in an older population [10].

The aims of this study were to 1) investigate the changes in frailty in a group of home-dwelling older adults above 70 years, after 8-year follow-up, 2) to investigate the association between dietary factors at baseline (protein intake from different dietary sources) and change in FI after 8-year follow-up and 3) the change in dietary intake with change in FI after 8-year follow-up.

Our hypotheses were that 1) increased rate of frailty with advancing age, 2) a higher intake of dietary protein at baseline is associated with a lower change in frailty after 8 years (more protein means less frail), and 3) an inverse association between change in dietary protein and change in frailty, where an increase in dietary protein intake is associated with a lower change in frailty (less frail), while a decrease in dietary protein intake is associated with a bigger change in frailty (more frail) after 8 years.

## 2 Methods

### 2.1 Study population and design

**Fig. 1:**
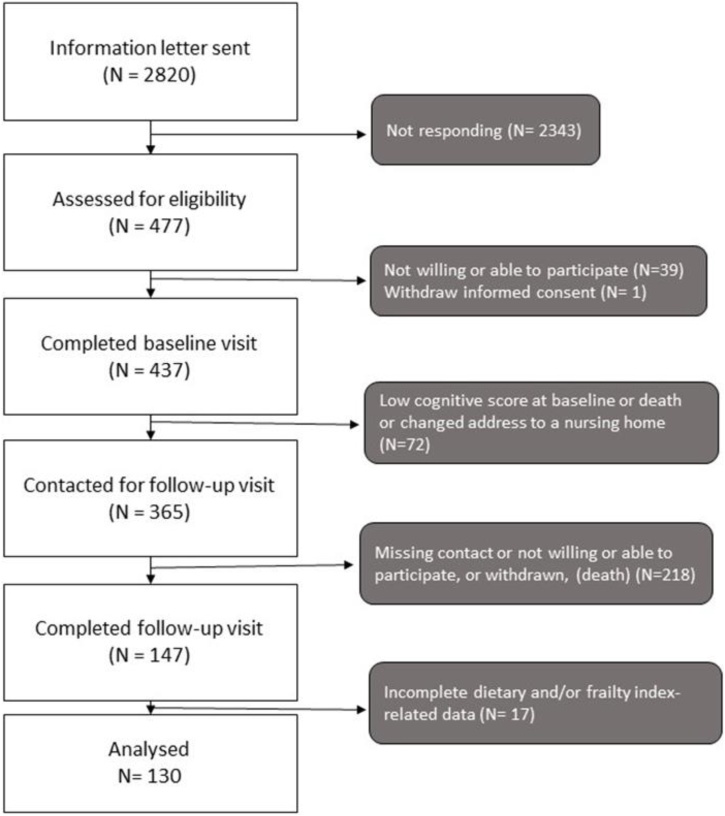
Flow chart of recruitment and inclusion process from 2014 to 2023.

The present study was a longitudinal study, collecting data at two time points. Home-dwelling men and women aged ≥ 70 years, living in South-East Norway were recruited through the National Population Register in 2014/2015, as described previously [11,12]. Briefly, a total of 2820 subjects were invited, and 437 subjects participated in the first visit. In 2022, we invited those who were still home-dwelling for an 8-year follow-up visit. A new extraction was made from the National Population Register for the same people to check if they were still home-dwelling. In total, 147 subjects participated in the follow-up study (Fig. 1), but only 130 participants had data available to calculate the FI score and dietary intake at two time points and were therefore included in the present analysis. The study was conducted according to the guidelines in the Declaration of Helsinki and approved by the Regional Committees for Medical and Health Research Ethics, Health Region South East, Norway (107167/REC). Written informed consent was obtained from the participants.

### 2.2 Frailty index (FI)

FI was constructed based on the standard procedure by Searle et al. [5] and has previously been described [11,13]. Thirty-eight deficits were included in the FI, where the scores of the 38 deficits were summarised and divided by the total number of deficits to yield a proportion [5]. Missing variables ≥ 20% of the population were excluded from the analyses (n = 0 in this sample). This resulted in a continuous FI scale, scored between 0 and 1. We also categorised frailty according to previous studies, defining frail participants (FI score ≥ 0.25) and non-frail participants (FI score < 0.25) [[14], [15], [16], [17], [18]].

### 2.3 Dietary assessment

Data on dietary intake were collected by 2 × 24-h recalls at each study visit, performed by trained dietitians as previously described [12]. The first visit was a face-to-face interview, while the second was an unannounced telephone interview 2–3 weeks after the study visit. The interview was conducted in a three-step process as previously described [19].

In this study, our focus was on the intake of protein from different dietary sources (total protein intake in grams, grams/kg BW, energy percent (E %), animal and plant protein, as well as the protein rich food groups “fish and seafood” (grams), “meat and meat products” (grams), and “dairy products” (grams)).

### 2.4 Routine markers

Non-fasting venous blood samples were collected by trained bioengineers at the study visit, as previously described [12].

### 2.5 Statistical analysis

Normally distributed data were presented as mean with standard deviation (SD), non-normally distributed data were presented as median with 25–75 percentiles. Categorical variables were presented as frequencies (n) and relational proportions (%). For continuous variables, paired t-tests and Wilcoxon signed ranks test were used on normally distributed and non-normally distributed data, respectively. McNemar’s test was used to compare paired categorical data. Changes in dietary intake and changes in FI score were calculated according to values at follow-up minus values at baseline. Correlations were tested by using Spearman’s rank correlation coefficient. Linear regression analyses were used to investigate the effect of nutrient intake at baseline on changes in FI during the follow-up (unadjusted and adjusted for age, sex, smoking, education, and fat mass in percent at baseline). Right-skewed data were log-transformed to fit the model, and a constant was added to avoid problems with zero intake of certain foods.

## 3 Results

### 3.1 Population characteristics

The population characteristics of the 130 home-dwelling older women (48%) and men (52%) included in the present study are presented in Table 1. After 8-year follow-up, more subjects were living alone, and the participants had lower MMSE-NR, MNA, SPPB scores, as well as lower hand grip strength after 8-year follow-up. More subjects had cardiovascular disease and used more drugs at the follow-up visit (Table 1).

**Table 1:** Characteristics of the study population, at baseline and follow-up.

	Baseline (N = 130)	Follow-up (N = 130)	P-value
Female*, n (%)*	63 (48.5)	63 (48.5)	
Age, *years*	73 (71, 75)	80 (79, 83)	
BMI, *kg/m*^2^	26.2 ± 3.5	26.0 ± 3.7	0.24
Waist, *cm*	96 ± 11	93 ± 12	**<0.001**
Hips, *cm*	103 ± 7	102 ± 8	**0.001**
FI score	0.12 ± 0.07	0.2 ± 0.11	**<0.001**
Frail*, n (%)*	4 (3.1)	43 (33.1)	**<0.001**
Single household*, n (%)*	38 (29.2)	48 (36.9)	**0.03**
MMSE-NR score	30 (26, 30)	29 (27, 29)	0.11
*< 24 points,* n (%)	0 (0)	4 (3.1)	***0.05***
MNA score	28.5 (27.5, 29)	28 (26.5, 28.5)	**<0.001**
*Non-malnourished (>23.5p), n (%)*	129 (99.2)	125 (96.2)	***0.05***
*Risk of malnutrition (17-23.5p), n (%)*	1 (0.8)	5 (3.9)	***0.05***
*Malnourished (<17p), n (%)*	0 (0)	0 (0)	
SPPB score	12 (11, 12)	11 (10, 12)	**<0.001**
*≤ 8 points, n (%)*	0 (0)	12 (9.2)	**<0.001**
Grip strength (kg), *dominant*	31.4 ± 9.8	27.4 ± 9.5	**<0.001**
Current smoker*, n (%)*	5 (3.9)	3 (2.3)	0.16
CVD*, n (%)*	27 (20.7)	61 (46.9)	**<0.001**
Diabetes*, n (%)*	7 (5.4)	11 (8.5)	0.10
Polypharmacy (≥5 drugs/day), *n (%)*	14 (10.8)	47 (35.2)	<0.001
Blood values			
Serum 25-OH-vitamin D3*, nmol/l*	84 ± 25.5	82.3 ± 23.1	0.43
Plasma Hb, g/100ml	14.3 ± 1.1	13.7 ± 1.20	**<0.001**
Serum albumin*, g/l*	41.6 ± 2.2	41.1 ± 2.4	**<0.01**
Serum creatinine*, μmol/L*	76 (66, 89)	80 (67, 92)	**<0.01**
Serum total cholesterol, *mmol/l*	5.3 ± 1.0	4.9 ± 1.2	**<0.001**
Serum LDL-cholesterol, *mmol/l*	3.2 ± 0.9	3.0 ± 1.2	**0.04**
Serum HDL-cholesterol, *mmol/l*	1.6 ± 0.5	1.6 ± 0.5	0.5
Serum TG, *mmol/l*	1.3 (0.98, 1.93)	1.24 (1.03, 1.62)	0.20
Serum C- reactive protein*, *mg/l*	1.5 (0.8, 2.9)	0.9 (0.5, 1.8)	**<0.001**
High Plasma HbA1c **, n (%)	9 (6.9)	10 (7.7)	0.74

SD, Standard deviation; MMSE-NR, Mini Mental Status Evaluation-Norwegian version; MNA, Mini Nutritional Assessment; SPPB, Short Physical Performance Battery; CVD, Cardiovascular Disease; Hb, Hemoglobin; LDL, Low-density lipoprotein; HDL, High-density lipoprotein; TG, Triglycerides; FI, Frailty Index. Normally distributed data is presented as mean (SD), non-normally distributed data is presented as median (25, 75 percentile). P-values: Continuous normally distributed data were tested by paired t-test, and continuous non-normally distributed data were tested by Wilcoxon sign rank test. McNemar's test was used for categorical data. Statistical significant level: P-value <0.05.

* Baseline: CRP < 50 mg/l included in the analyses (N = 132). Follow-up: measured as microCRP.

** Def: ≥6,5% for Baseline, and ≥48 mmol/mol for Follow-up.

### 3.2 Frailty index

Compared to baseline, after 8-year follow-up, the mean FI score in the study population was significantly higher, and the percentage of older subjects categorised as frail had increased from 3% at baseline to 33% at follow-up (Table 1). Interestingly, 8% (n = 10) of the study population had a decrease in FI score (improved FI) and were less frail at follow-up. In contrast, 10% (n = 13) had an increase > 0.2 in FI score from baseline to follow-up (more frail) **(Data not shown)**. More men were represented in the group with an improved FI score (70% men), while women dominated the group with a decline in frailty (increased FI score; 69% women). More subjects were living alone at both visits in the more frail group compared to the less frail group).

### 3.3 Biochemical markers

More subjects used medications at follow-up (e.g, cholesterol-lowering and anti-inflammatory medications), leading to improved TC, LDL-cholesterol, and CRP levels. More biochemical changes are shown in Table 1.

### 3.4 Dietary intake

The mean protein intake (E %) was significantly lower at follow-up compared to baseline. No statistically significant differences in absolute intake of protein, grams of protein per kg BW, nor in the intake of animal protein or plant protein were seen between the two visits (Table 2 for all). For the protein sources, the intake of meat was significantly decreased from baseline to follow-up (**Data not shown**).

**Table 2:** Dietary intake of macronutrients.

	Baseline (N = 130)	Follow-up (N = 130)	P-value
Energy, *kJ*	7721(6346, 8988)	7859 (6654, 9334)	0.13
Protein, *g*	80.9 ± 23.3	78.3 ± 23.6	0.18
Protein*, g/kg BW*	1.09 ± 0.4	1.08 ± 0.37	0.76
Protein, *E %*	17.9 ± 4	16.8 ± 3.6	**<0.01**
Animal protein, *g*	50.2 ± 18.9	53.7 ± 20.7	0.12
Plant protein, *g*	20.5 (15.1, 27.2)	19.4 (15.5, 25.5)	0.34
Total fat*, E %*	37.1 ± 7.8	39.3 ± 7.0	**<0.01**
Saturated fat, *E %*	14.4 ± 4.1	15.1 ± 4	**0.06**
Polyunsaturated fat*, E %*	6.3 (4.8, 8.0)	6.3 (5.3, 7.7)	0.84
Monounsaturated fat, *E %*	12 (10.1, 13.9)	14.1 (12.0, 16.0)	**<0.001**
Carbohydrates, *E %*	40.4 ± 7.5	40.0 ± 7.3	0.62
Added sugar, *E %*	5.8 (3.8, 8.8)	5.9 (4.1, 8.4)	0.43
Fiber, *E %*	2.2 (1.8, 2.6)	2.0 (1.7, 2.5)	0.37
Fiber, *g*			
Women	19.2 ± 6.8	19.8 ± 5.7	0.52
Men	22.3 ± 7.6	22.2 ± 9.0	0.88
Alcohol, *E %*	0 (0, 4.0)	0 (0, 2.3)	0.11

SD, Standard deviation; KJ, Kilo Joule; kg, kilograms; g, grams; E %, percent of total energy intake. Normally distributed data presented as mean (SD), non-normally distributed data are presented as median (25, 75 percentile). P-values: Continuous normally distributed data were tested by paired t-test, and continuous non-normally distributed data were tested by Wilcoxon sign rank test. Statistically significant level: P-value <0.05.

### 3.5 Dietary factors and change in FI

**Fig. 2:**
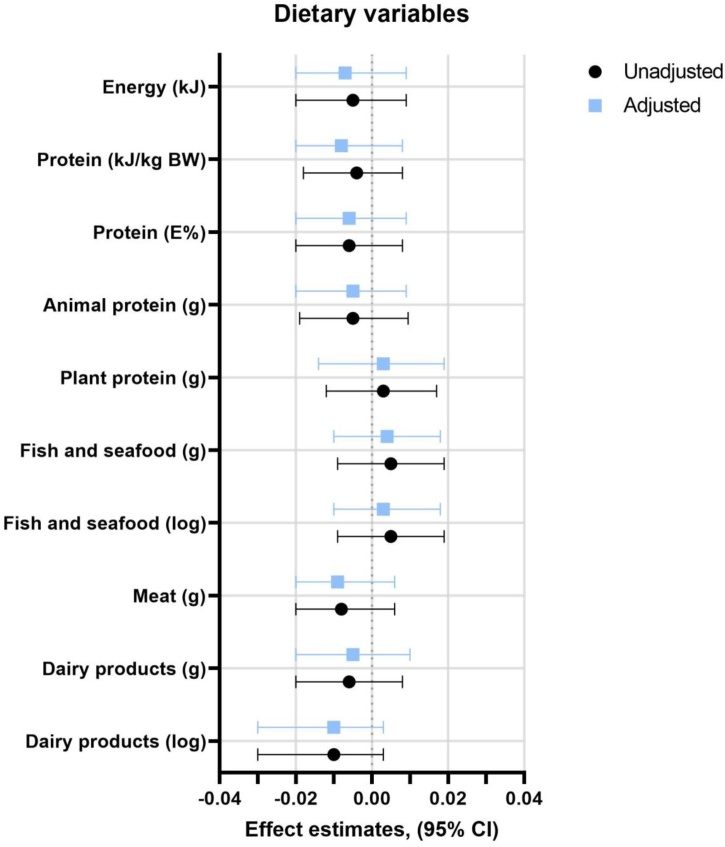
Forest plot of associations between dietary variables at baseline (exposure) and changes in FI score from baseline to follow-up (outcome). The changes in the FI score were calculated by taking the FI score at the follow-up visit minus the FI score at the baseline visit. Due to the different scales of the dietary variables, we normalized all dietary variables to z-scores to visualize our results. The z-score was calculated for each dietary variable by subtracting the mean and dividing by the standard deviation. The linear regression models were calculated as described in Methods. The adjusted linear regression model was adjusted for sex, age, smoking and fat mass (in percent) at baseline.

We investigated whether dietary intake at baseline could explain the change in FI after 8-year follow-up. No associations between dietary factors at baseline and change in FI from baseline to follow-up (adjusted and non-adjusted), no correlation between change in dietary factor and change in FI from baseline to follow-up (Fig. 2).

## 4 Discussion

In the present study, we showed a significant increase in frailty after 8-year follow-up, but no significant association between our dietary markers and change in FI after 8-year follow-up.

Overall, the study population participating in the follow-up study was relatively healthy and had a lower FI score than the total study population who participated at baseline [20]. Still, we showed a significant increase in FI score after 8-year follow-up. Moreover, the number of subjects categorised as frail increased 10-fold during the 8-year follow-up, suggesting that old subjects are particularly susceptible to a decline in health in this period.

Previous studies have shown that FI score is positively associated with age [[21], [22], [23], [24]], and women are more frail than men [17]. In our study, the participants' median age changed from 73 years at baseline to 80 years at follow-up. This transition period seems to be crucial for health changes. At 8-year follow-up, more participants were categorised to have a low score of physical function and lower grip strength compared to the baseline visit. Even though there were minor changes in dietary intake between the two visits, we showed a significant decrease in MNA score, reflecting a trend toward a higher risk of malnutrition.

In the 8-year follow-up period, we found a significant decrease in the intake of protein (E %) and in the consumption of meat (grams). There was no significant change in the intake of fish and seafood or dairy products between baseline and follow-up. We found no association between protein intake at baseline or changes in protein intake and changes in FI. The recommended intake of protein in older adults is 1.2–1.5 g/kg body weight per day, or 15–20 E % protein per day. In the present study, the protein intake was slightly lower than the recommended level, while the intake in E % met the recommendation.

An 11-year follow-up from the Rotterdam Study showed a positive association between isocaloric intake of 10 g protein and increased frailty over time (mainly driven by animal protein) [25]. In contrast, studies have shown conflicting data on protein intake and association to frailty [[26], [27], [28]],

Results from the Tromsø study, investigating older Norwegian adults, showed that a higher intake of proteins (in g/kg) was associated with a lower risk of pre-frailty and frailty, by using Fried’s definition [29].

We showed no significant associations between protein source (animal or plant protein) and change in FI. Studies have shown positive association, negative or no association between animal protein and frailty [[25], [26], [27]]. However, studies have shown a significant negative association between an intake of plant protein and risk of physical frailty [30,31]. In our study population, animal protein was the main protein source. The overall low intake of plant protein in our study population makes it difficult to compare the effect of animal versus plant protein sources on the development of frailty.

The intake of meat decreased significantly from baseline to follow-up, however, no association between consumption of meat at baseline or change in intake and change in FI was found. The Nurses’ Health Study showed an association between the consumption of red meat (processed and unprocessed) and increased risk of physical frailty [32]. In contrast, a Japanese study showed that an increased intake of meat was associated with a lower risk of physical frailty [33].

We found no change in fish and seafood intake after 8 years, no significant associations between baseline intake of fish and seafood or changes in fish and seafood intake and changes in FI score from baseline to follow-up [10]. Increased seafood consumption (fatty fish, EPA, and DHA) has been associated with a lower accumulation of age-related deficits [10] a high intake of fish (both lean, fatty, and total fish) reduced the risk of being pre-frail in a group of old subjects [34].

We did not find any changes in the intake of dairy products between the two study visits. There was no significant association between the intake of dairy products at baseline and the change in dairy intake and the change in FI score in the 8-year follow-up. As far as we know, our study is the first to investigate the association between dairy products and FI. Studies on dairy products and Fried’s frailty definition show inconsistent results; two studies have shown an association between consumption of dairy products and reduced the risk of physical frailty [35,36]. In contrast, longitudinal studies investigating dairy products and physical frailty in older adults showed no significant association between consumption of dairy products and risk of frailty [31,37]. Ottestad et al. [38] investigated the effect of protein-enriched milk on physical function and strength in a randomised controlled trial. No significant improvement in physical function or muscle strength was shown in the healthy adults who received protein-enriched milk, compared to the isocaloric carbohydrate-drinking control group [38].

### 4.1 Strengths and limitations

The strength of our study is the longitudinal design, use of validated methods, and use of repeated measurements.

The present study also has some limitations. Only a small part of the total study population from 2014/2015 was included in this study (30%). The study population is small, which limits the the generalizability to the general population aged ≥ 70 years. The low FI score in the present study population at baseline suggests that only the healthiest older adults participated in the study.

To collect dietary data, we used 2 × 24-h recall per visit, which may influence the possibility of comparing dietary intake between studies. The follow-up study was conducted right after the COVID-19 pandemic, which may have influenced activity levels and quality of life and feelings of loneliness [39,40].

## 5 Conclusions

In the present study, we found that healthy home-dwelling older adults were more frail after eight years of follow-up. In the present study, we found no association between dietary factors at baseline and change in FI score, or between change in dietary factors and change in FI score after eight years. More studies are needed to define what factors might accelerate or prevent frailty.

## Authors' contributions

Conceptualization: P.B, SMU, KBH; Data Curation: PB, MT, IO, KR; Formal Analysis: PB, MT, JJC; Funding acquisition: SMU, KBH, AH; Investigation: PB, SMU, KBH, KR, US; Methodology: PB, SMU, KBH, MT, AH, IO, US; Project administration; SMU, KBH; Supervision: SMU, KBH; Visualization: PB, JJC; Writing original draft: PB, SMU, KBH; Writing, review & editing: all authors.

## Ethics approval and consent to participate

The study was conducted according to the guidelines in the Declaration of Helsinki and approved by the Regional Committees for Medical and Health Research Ethics, Health Region South East, Norway (107167/REC). Written informed consent was obtained from the participants. Extracts from the National Population Registry were used according to, and with approval from, the Norwegian Tax Administration.

## Consent for publication

Not applicable.

## Declaration of Generative AI and AI-assisted technologies in the writing process

During the preparation of this work the author(s) used ChatGPT in order to correct language in specific sentences. After using this tool/service, the author(s) reviewed and edited the content as needed and take(s) full responsibility for the content of the published article.

## Funding

The present study was supported by grants from the Throne Holst Foundation for nutrition research, University of Oslo.

## Data availability

The datasets used and/or analysed during the current study are available from the corresponding author on reasonable request.

## Declaration of competing interest

During the past 5 years, K.B.H. has received honoraria from Sanoofi, Amgen, Menarini and Ultragenyx, none of which are related to the contents of this manuscript. PB, SMU, MT, US, KR, AH, JJC, and IO have no conflict of interest.
